# Effect of uric acid and estimated glomerular filtration rate on the Fibrosis-4 index: A cross-sectional and cohort analysis

**DOI:** 10.1097/MD.0000000000051217

**Published:** 2026-10-16

**Authors:** Weina Xu, Danxiang Chen, Saiyun Lou, Jiannan Wu, Yinshan Liu, Ruiqi Liu, Hanjie Chen

**Affiliations:** a Department of General Medicine, Fourth Affiliated Hospital, Zhejiang University School of Medicine, Yiwu City, Zhejiang Province, China; b Department of Geriatric Medicine, Center for Regeneration and Aging Medicine, International Institutes of Medicine, Zhejiang University, Yiwu City, Zhejiang Province, China; c Faculty of Information, University of Toronto, Toronto, ON, Canada; d Department of Information Technology, Fourth Affiliated Hospital, Zhejiang University School of Medicine, Yiwu City, Zhejiang Province, China.

**Keywords:** chronic kidney disease, estimated glomerular filtration rate, Fibrosis-4 index, liver fibrosis, uric acid

## Abstract

Hepatic steatosis and fibrosis present a significant public health burden, especially in China. Emerging evidence suggests an association between liver fibrosis and kidney function, indicating a potential relationship among uric acid (UA) levels, estimated glomerular filtration rate (eGFR), and the Fibrosis-4 (FIB-4) index. This retrospective cohort study investigates the correlation between UA, estimated GFR, and FIB-4 index in Chinese adults with metabolic dysfunction-associated steatotic liver disease (MASLD) using cross-sectional and longitudinal analyses. A total of 5051 adults with MASLD were included from 8857 individuals initially screened at the International Healthcare Center, Fourth Affiliated Hospital, Zhejiang University School of Medicine. The main outcome measure was the FIB-4 index. Among the 5051 participants (75.4% male; mean age, 42.3 years), the mean baseline eGFR was 98.7 mL/min/1.73 m^2^. Participants with lower eGFR had significantly older age and higher body mass index, systolic blood pressure, triglycerides, UA levels, and FIB-4 index than those with higher eGFR (*P* < .001 for all). In multivariable Cox regression analyses, each 1-standard deviation decrease in eGFR was associated with a 21% higher risk of an increased FIB-4 index (hazard ratio [HR], 1.21; 95% confidence interval [CI], 1.14–1.29; *P* < .001). Hyperuricemia (UA ≥ 420 μmol/L) was independently associated with a 38% higher risk of elevated FIB-4 (HR, 1.38; 95% CI: 1.23–1.55; *P* < .001). In the unadjusted model, a significant nonlinear dose-response relationship was observed between eGFR and FIB-4 (*P* for nonlinear trend < .001). However, after progressive adjustment for demographic variables, family history, and metabolic covariates, the nonlinear signal was markedly attenuated. Mediation analysis demonstrated that UA partially mediated the association between eGFR and FIB-4, accounting for 6.88% of the total effect. Reduced eGFR and hyperuricemia were independently associated with an elevated FIB-4 index in adults with MASLD, with a nonlinear relationship indicating a greater liver fibrosis risk in individuals with moderate to severe renal impairment (eGFR < 60 mL/min/1.73 m^2^). Partial mediation by UA suggests that metabolic dysregulation contributes to the kidney-liver interaction. Routine assessment of eGFR and UA may improve liver fibrosis risk stratification in patients with MASLD.

## 1. Introduction

The global burden of liver fibrosis, a pivotal pathological process underlying metabolic liver diseases, has risen steadily, particularly in China, where hepatic steatosis affects over 29% of adults and is closely associated with obesity (16.4%), type 2 diabetes (11.9%), and dyslipidemia (35.6%).^[1-3]^ Chronic kidney disease (CKD) affects approximately 10.8% of the Chinese population, and its frequent coexistence with these metabolic disorders has intensified scientific interest in the so-called “liver–kidney axis.” Metabolic dysfunction-associated steatotic liver disease (MASLD) and CKD are closely interconnected, and their intrinsic relationship is mediated by multiple convergent molecular pathways. Among these, inflammatory responses, oxidative stress, and fibrotic cascades constitute core pathophysiological mechanisms shared by both conditions, thereby reinforcing the biological basis of liver–kidney crosstalk.^[4,5]^ Renal-derived factors, particularly aberrant activation of the renin–angiotensin–aldosterone system (RAAS), have been shown to influence the development and progression of MASLD. Emerging evidence indicates that steatotic hepatocytes release pro-ferroptotic factors, such as free fatty acids and β-hydroxybutyrate, via paracrine signaling, thereby promoting abnormal Fe^2+^ accumulation in renal tubular epithelial cells and consequently inducing ferroptosis in the kidney. In metabolic dysfunction-associated steatohepatitis (MASH)-related renal injury, local lipid deposition and oxidative stress act synergistically to further potentiate ferroptotic pathway activation, establishing a vicious cycle between hepatic and renal injury.^[6]^ UTX (ubiquitously transcribed tetratricopeptide repeat on chromosome X), also known as KDM6A, is an H3K27 demethylase involved in the regulation of diverse biological processes. Recent studies have suggested that renal serine synthesis is regulated by UTX and may exert a significant impact on systemic metabolic homeostasis, particularly in hepatic metabolism. Notably, oral serine supplementation has been shown to effectively alleviate high-fat diet-induced fatty liver and renal dysfunction, suggesting its therapeutic potential for obesity-related metabolic disorders.^[7]^ Collectively these findings highlight how renal dysfunction, reflected by a reduced estimated glomerular filtration rate (eGFR), may exacerbate liver fibrosis, and vice versa, through mechanisms yet to be elucidated in large-scale population studies.^[8]^

Uric acid (UA), an important metabolite involved in metabolic homeostasis, has emerged as a key mediator of this inter-organ relationship.^[9]^ Hyperuricemia, which affects 17.7% of Chinese adults, is significantly associated with both reduced eGFR and advanced liver fibrosis. This association is thought to arise from UA-induced hepatic stellate cell activation and subsequent extracellular matrix deposition.^[10]^ However, current evidence remains limited by small sample sizes, short follow-up periods, and the underrepresentation of East Asian populations, whose metabolic risk profiles and CKD epidemiology differ markedly from those of Western populations. Moreover, the independent and combined effects of eGFR and UA on liver fibrosis markers, as well as the influence of metabolic comorbidities on these associations, remain unclear.^[11-13]^

Therefore,^[14,15]^ we conducted a comprehensive cross-sectional and cohort analysis in a large Chinese health-screening population to delineate the complex relationship between eGFR, UA, and the Fibrosis-4 (FIB-4) index. By integrating data from 5051 participants with a mean follow-up of 12.6 months, this study aimed to evaluate the nonlinear association between renal function and liver fibrosis, investigate the role of UA in the liver–kidney axis, and identify subpopulations that may be susceptible to these interactions.

Overall, this study sought to bridge critical translational gaps in our understanding of the liver–kidney axis and provide essential insights that may facilitate the development of integrated screening strategies to improve the prognosis of metabolic liver and kidney diseases.^[16–18]^

## 2. Materials and methods

### 2.1. Study design and participants

This cross-sectional cohort study analyzed data from China’s liver fibrosis screening and risk assessment initiative for high-risk populations (www.chpf.cn). Comprehensive demographic information, physical examination results, laboratory findings, and questionnaire responses were collected. A total of 8857 participants were initially included. After excluding individuals with missing sociodemographic data at baseline or during follow-up, as well as those with missing data on body mass index (BMI), triglyceride (TG), fasting blood glucose (FBG), low-density lipoprotein cholesterol (LDLC), UA, or parental hypertension (HT) history, 5051 participants remained for the final analysis (Fig. 1). This study was approved by the Ethics Committee of the Fourth Affiliated Hospital of Zhejiang University School of Medicine. Written informed consent was obtained from all participants before enrollment.

**Figure 1.**
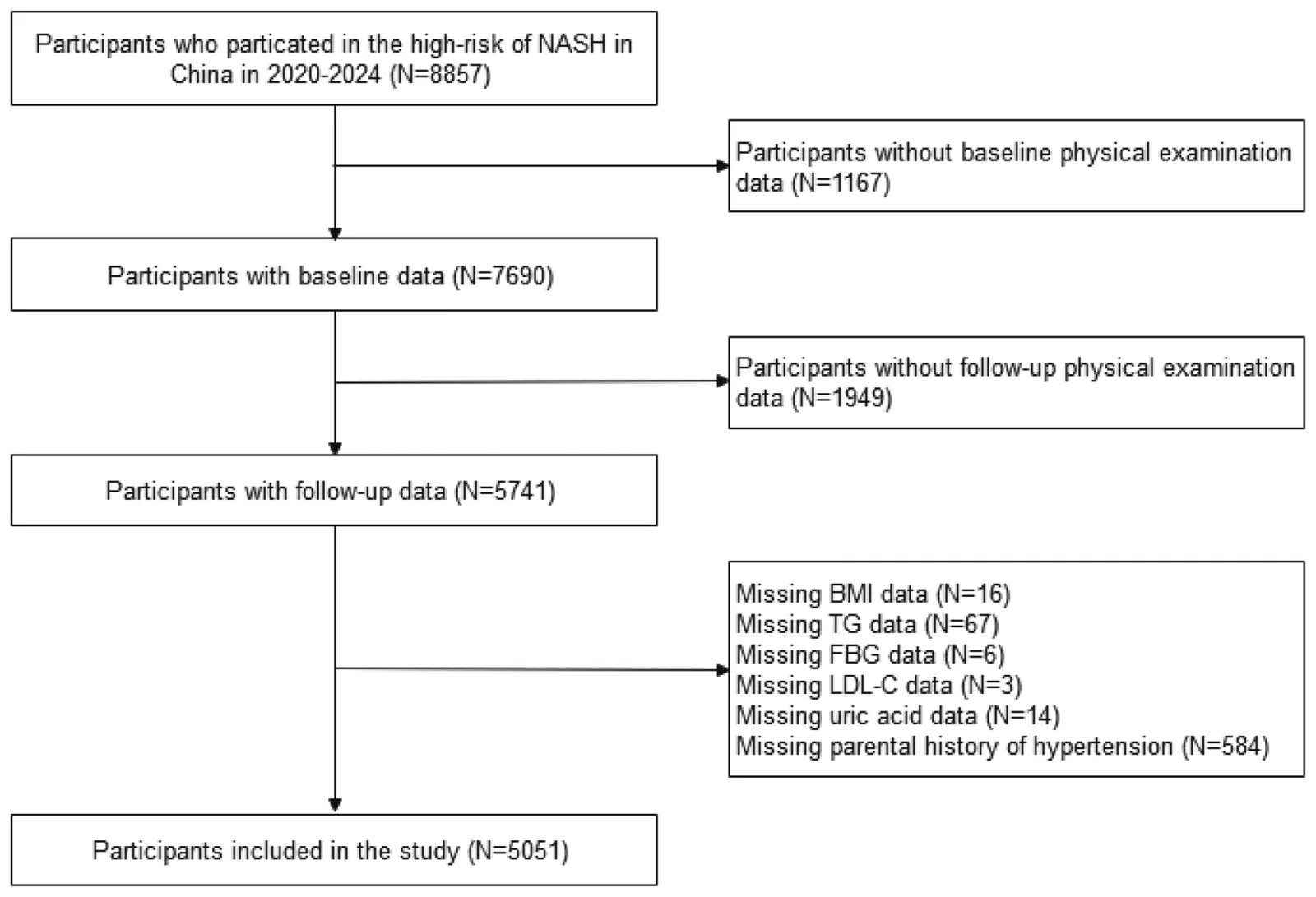
Participant flowchart for the study. In the 2020–2024 Chinese cohort study of individuals at high risk for nonalcoholic steatohepatitis, 8857 participants were initially screened. Of these, a total of 1167 participants were excluded due to missing baseline physical examination data, leaving 7690 participants with complete baseline assessments. A total of 1949 participants were excluded because they lacked follow-up physical examination data, resulting in 5741 participants with available data. Finally, participants with missing data on the following key covariates were excluded: BMI (n = 16), TG (n = 67), FBG (n = 6), LDL-C (n = 3), UA (n = 14), and parental history of hypertension (n = 584). After sequential exclusion, 5051 participants were included in the final analysis. BMI = body mass index, eGFR = estimated glomerular filtration rate, FBG = fasting blood glucose, LDL-C = low-density lipoprotein cholesterol, TG = triglyceride, UA = uric acid.

### 2.2. Data collection and definition

Demographic information, including sex, age, and parental HT history (categorized as neither parent affected or at least 1 parent affected), was collected for all participants. Fasting blood samples collected during health examination visits were used to measure alanine aminotransferase (ALT), aspartate aminotransferase (AST), platelet count (PLT), triglycerides (TG), total cholesterol (TCHOL), high-density lipoprotein cholesterol (HDLC), LDLC, FBG, UA, and serum creatinine (SCr). Age, BMI, systolic blood pressure (SBP), diastolic blood pressure (DBP), ALT, AST, PLT, TG, TCHOL, HDLC, LDLC, FBG, UA, SCr, and the FIB-4 index were analyzed as continuous variables. The mean follow-up duration was 12.6 ± 5.5 months.

### 2.3. Exposure variables and outcomes

The primary exposure variables were UA level and eGFR. UA levels were categorized into 2 groups: <420 µmol/L (normal group) and ≥ 420 µmol/L (hyperuricemia group). The CKD-EPI 2021 creatinine-based equation, recommended by the Kidney Disease: Improving Global Outcomes (KDIGO), was used to estimate eGFR. eGFR, which represents the per minute volume of plasma filtered by the glomeruli, serves as a crucial indicator for measuring kidney function.

CKD-EPI 2021 Creatinine-Based Equation^[19]^:


$$\begin{array}{c} eGFR=142\times \min{\left(Scr/\kappa ,1\right)}^{\alpha }\times \max{\left(Scr/\kappa ,1\right)}^{-1.200}\\ \times 0.9938^{Age}\times 1.012\left[if\;female\right] \end{array}$$

This formula incorporated several key parameters: SCr measured in milligrams per deciliter (mg/dL), a sex-specific constant κ (0.7 for females and 0.9 for males), an exponent α (−0.241for females and −0.302 for males); min indicates the minimum of Scr/κ or 1, max indicates the maximum of Scr/κ or 1.

Age and sex were also included in the calculation, with multipliers of 1.018 for females and 1 for males. The FIB-4 index, first introduced by Sterling in 2006 as a noninvasive tool for assessing liver fibrosis, is well suited for screening liver fibrosis in the general population during routine physical examinations.^[20]^


$$\begin{array}{c} The\;calculation\;formula\;is:FIB-4=\left(Age\times AST\right)\\ ÷\left(PLT\times \sqrt{ALT}\right). \end{array}$$

### 2.4. Statistical analysis

Continuous variables are presented as mean ± SD, and categorical variables as frequencies (percentages). The Kolmogorov–Smirnov test was used to assess the normality of continuous data. To streamline analysis and align with the KDIGO 2021 classification, eGFR was categorized into 3 groups: ≥90, 60 to 89, and <60 mL/min/1.73m^2^; the latter encompassed stages G3a–G5.^[21]^ Normally distributed continuous variables compared using independent *t* tests, whereas non-normally distributed variables were presented as medians and interquartile ranges (IQR, 25–75th percentiles) and analyzed using Wilcoxon rank-sum tests. Categorical variables were assessed using Pearson chi-squared test. The FIB-4 index was classified as <2.68 or ≥2.68 using the validated threshold for advanced liver fibrosis.^[22]^ Derived from a multi-etiology cohort study, this cutoff, determined by maximizing Youden index, demonstrated robust performance with a sensitivity of 70.7% and a specificity of 79.1% for detecting advanced fibrosis across various liver diseases.^[23]^ Values ≥2.68 indicate a significant risk for hepatic fibrosis, consistent with prior validation studies, and warrant further clinical evaluation.

Using this framework, we develop 3 distinct analytical models. Model 1 included eGFR only, model 2 augmented model 1 by adjusting for age, sex, UA level, SBP, and BMI, and model 3 further adjusted for TG, TCHOL, and FBG, and parental HT status (categorized as neither parent hypertensive vs at least 1 parent hypertensive).

To investigate the risk factors associated with the FIB-4 index, restricted cubic spline (RCS) curves were used to explore the potential nonlinear relationship between eGFR and the FIB-4 index. After categorizing the population according to sex, age, parental history of HT, FBG, SBP, TCHOL, TG, UA, and BMI, subgroup analyses were conducted using Cox proportional hazards models.

Subsequently, mediation analysis was performed to examine the indirect and direct pathways linking eGFR to the FIB-4 index, with UA acting as the mediator. Within this framework, eGFR functioned as the predictor variable (X), UA as the mediator variable (M), and the FIB-4 index as the outcome variable (Y). RCS modeling was performed using the “rms” package, and mediation analysis was conducted using the “mediation” package.^[24,25]^ Statistical significance was defined as *P* < .05. All statistical analyses were performed using R version 4.4.3 (https://mirrors.zju.edu.cn/CRAN).

## 3. Results

### 3.1. Participant baseline characteristics

This study included 5051 participants (75.4% male; mean age: 42.3 years), who were categorized according to eGFR levels (Table 1). The cohort included 4050 individuals (80.2%) with eGFR ≥ 90 mL/min/1.73m^2^ (eGFR1), 971 (19.2%) with eGFR of 60 to 89 mL/min/1.73m^2^ (eGFR2), and 30 (0.6%) with eGFR < 60 mL/min/1.73m^2^ (eGFR3). Age increased progressively with declining eGFR groups (*P *< .001), with half of the participants in the eGFR3 group aged ≥ 60 years. The proportion of males also increased significantly, from 73.0% in eGFR1 to 96.7% in eGFR3 (*P *< .001). BMI, SBP, and TG showed a stepwise increase across the eGFR groups (all *P *≤ .002), whereas LDLC was significantly lower in the eGFR3 group (*P *< .001). The prevalence of fasting hyperglycemia (FBG ≥ 7.0 mmol/L) was significantly higher in the eGFR3 group (*P *< .001). SCr and UA concentrations progressively increased with decreasing eGFR (both *P *< .001), whereas PLT levels decreased (*P *< .001). The FIB-4 index was highest in the eGFR3 group (*P *< .001), and 6.7% of the participants had a FIB-4 index ≥ 2.68 (*P *< .001). Overall, lower eGFR was associated with older age, a higher proportion of males, adverse metabolic characteristics, and higher FIB-4 index values. Although the small eGFR3 sample size (n = 30) limited definitive conclusions regarding moderate-to-severe renal insufficiency, the observed trend across continuous variables remained consistent.

**Table 1:** Baseline characteristics of the study population across different eGFR levels.

Characteristic	Overall	eGFR (≥90 mL/min/1.73m^2^)	eGFR (≥60 and <90 mL/min/1.73m^2^)	eGFR (<60 mL/min/1.73m^2^)	*P*-value
	(n = 5051)	(n = 4050)	(n = 971)	(n = 30)	
Age (in yr)	42 (34, 51)	39 (32, 49)	51 (44, 57)	60 (49, 69)	<.001
Age group, n (%)					<.001
<40	2212 (43.79)	2058 (50.81)	152 (15.65)	2 (6.67)	
40–60	2528 (50.05)	1859 (45.90)	656 (67.56)	13 (43.33)	
60+	311 (6.16)	133 (3.28)	163 (16.79)	15 (50.00)	
Sex, n (%)					<.001
Male	3807 (75.37)	2956 (72.99)	822 (84.65)	29 (96.67)	
Female	1244 (24.63)	1094 (27.01)	149 (15.35)	1 (3.33)	
BMI (kg/m^2^)	25.98 (24.13,28.01)	25.91 (24.04,28.00)	26.27 (24.49,28.05)	26.80 (25.04,29.28)	.002
BMI group, n (%)					<.001
<25	1861 (36.84)	1547 (38.20)	307 (31.62)	7 (23.33)	
≥25, <30	2663 (52.72)	2075 (51.23)	570 (58.70)	18 (60.00)	
≥30	527 (10.43)	428 (10.57)	94 (9.68)	5 (16.67)	
SBP (mm Hg)	125 (115,136)	124 (114,135)	128 (117,140)	139 (126,152)	<.001
SBP group, n (%)					
<140 mm Hg	4104 (81.25)	3374 (83.31)	715 (73.64)	15 (50.00)	<.001
≥140 mm Hg	947 (18.75)	676 (16.69)	256 (26.36)	15 (50.00)	
DBP (mm Hg)	76 (68,84)	76 (68,84)	78 (71,86)	76 (71,88)	<.001
DBP Group, n (%)					.004
<90 (mm Hg)	4331 (85.75)	3504 (86.52)	804 (82.80)	23 (76.67)	
≥90 (mm Hg)	720 (14.25)	546 (13.48)	167 (17.20)	7 (23.33)	
ALT (U/L)	28 (20, 43)	28 (19, 44)	28 (20, 39)	25 (18, 44)	.452
AST (U/L)	25 (21, 31)	25 (21, 31)	26 (22, 31)	25 (20, 33)	<.001
PLT (10^9^/L)	238 (205, 279)	243 (209, 284)	221 (191, 256)	200 (176, 239)	<.001
TG (mmol/L)	1.78 (1.29, 2.55)	1.75 (1.28, 2.51)	1.92 (1.35, 2.70)	2.19 (1.28, 3.29)	<.001
TG group					<.001
≤1.7 mmol/L	2353 (46.58)	1938 (47.85)	405 (41.71)	10 (33.33)	
>1.7mmol/L	2698 (53.42)	2112 (52.15)	566 (58.29)	20 (66.67)	
TCHOL (mmol/L)	5.17 (4.57, 5.81)	5.17 (4.59, 5.81)	5.17 (4.54, 5.86)	4.63 (3.49, 5.49)	.003
≤5.18 mmol/L	2551 (50.50)	2042 (50.42)	489 (50.36)	20 (66.67)	.207
>5.18 mmol/L	2500 (49.50)	2008 (49.58)	482 (49.64)	10 (33.33)	
HDLC (mmol/L)	1.08 (0.93, 1.25)	1.08 (0.94, 1.26)	1.06 (0.92, 1.23)	1.06 (0.85, 1.13)	.005
LDLC (mmol/L)	3.10 (2.62, 3.64)	3.11 (2.64, 3.65)	3.04 (2.57, 3.61)	2.30 (1.78, 3.00)	<.001
FBG (mmol/L)	4.79 (4.45, 5.20)	4.77 (4.43, 5.16)	4.89 (4.52, 5.41)	5.54 (4.84, 6.75)	<.001
FBG group					
<7 mmol/L	4818 (95.4)	3871 (95.58)	924 (95.16)	23 (76.67)	<.001
≥7 mmol/L	233 (4.6)	179 (4.42)	47 (4.84)	7 (23.33)	
SCr (mg/dL)	0.90 (0.70, 1.00)	0.80 (0.70, 0.90)	1.00 (1.00, 1.10)	1.50 (1.30, 1.60)	<.001
UA (μmol/L)	382 (324, 441)	377 (317, 434)	406 (355, 462)	408 (341, 491)	<.001
UA group, n (%)					
<420 μmol/L	3368 (66.68)	2796 (69.04)	555 (57.16)	17 (56.67)	<.001
≥420 μmol/L	1683 (33.32)	1254 (30.96)	416 (42.84)	13 (43.33)	
HT in parents, n (%)					<.001
Neither parent	2797 (55.38)	2294 (56.64)	492 (50.67)	11 (36.67)	
At least 1 parent	2254 (44.62)	1756 (43.36)	479 (49.33)	19 (63.33)	
FIB-4 index	0.83 (0.59, 1.18)	0.76 (0.56, 1.08)	1.14 (0.85, 1.51)	1.59 (1.20, 1.84)	<.001
FIB-4 group, n (%)					<.001
<2.68	4988 (98.75)	4017 (99.19)	943 (97.12)	28 (93.33)	
≥2.68	63 (1.25)	33 (0.81)	28 (2.88)	2 (6.67)	

With decreasing eGFR, participants showed significant trends of older age (*P* < .001), a higher male proportion (*P* < .001), increased BMI (*P* = .002), elevated blood pressure (SBP, *P* < .001; DBP, *P* < .001), and worse metabolic profiles, including higher TG (*P* < .001), FBG (*P* < .001), SCr (*P* < .001), and UA (*P* < .001). Marked differences were also observed in liver fibrosis markers (FIB-4 index, *P* < .001) and family history of hypertension (*P* < .001). Notable exceptions included alanine aminotransferase (ALT; *P* = .452), for which no significant intergroup differences were detected. These results highlight the association between reduced renal function and age-related metabolic and hemodynamic abnormalities in the study cohort.

ALT = alanine aminotransferase, AST = aspartate aminotransferase, BMI = body mass index, DBP = diastolic blood pressure, eGFR = estimated glomerular filtration rate, FBG = fasting blood glucose, FIB-4 = Fibrosis-4, HDLC = high-density lipoprotein cholesterol, HT = hypertension, LDLC = low-density lipoprotein cholesterol, PLT = platelet count, SBP = systolic blood pressure, SCr = serum creatinine, TCHOL = total cholesterol, TG = triglycerides, UA = uric acid.

### 3.2. Cox model analysis

Table 2 presents the correlation between eGFR and the FIB-4 index evaluated using Cox proportional hazards models. For continuous eGFR, each SD increase was significantly associated with a lower risk of the outcome across all models. In the unadjusted model (model 1), the hazard ratio (HR) was 0.60 (95% confidence interval [CI], 0.57–0.63; *P* < .0001). After adjustment for age, sex, UA level, and BMI (model 2), the HR was 0.79 (95% CI: 0.74–0.84; *P* = .0001). Further adjustment for parental HT, SBP, DBP, TG, TCHOL, and FBG (model 3) yielded a nearly identical HR of 0.79 (95% CI: 0.74–0.84; *P* = .0001), indicating that the association remained robust after adjustment for potential confounders.

**Table 2:** Hazard ratios for the primary outcome across estimated glomerular filtration rate quartiles (Q1–Q4, ordered from highest to lowest estimated glomerular filtration rate).

Characteristic	Participants	Model 1		Model 2		Model 3	
	n (%)	HR (95% CI)	*P*	HR (95% CI)	*P*	HR (95% CI)	P
Per SD increase		0.60 (0.57,0.63)	<.0001	0.79 (0.74,0.84)	.0001	0.79 (0.74,0.84)	.0001
Q1	1205 (23.86)	Ref		Ref		Ref	
Q2	1264 (25.02)	0.80 (0.71,0.91)	.0005	0.99 (0.87,1.12)	.8246	0.99 (0.87, 1.13)	.8753
Q3	1226 (24.27)	0.38 (0.33,0.45)	<.0001	0.59 (0.50,0.70)	.0001	0.59 (0.49, 0.70)	<.0001
Q4	1356 (26.85)	0.09 (0.07,0.12)	<.0001	0.23 (0.17,0.31)	<.0001	0.23 (0.17, 0.31)	<.0001
*P* for trend		<.0001		<.0001		<.0001	

Data are presented as hazard ratios (HRs) with 95% CIs estimated using Cox proportional hazards models. Estimated glomerular filtration rate (eGFR) quartiles were categorized according to the distribution of estimated glomerular filtration rate values: Q1 (highest estimated glomerular filtration rate, reference), Q2, Q3, and Q4 (lowest estimated glomerular filtration rate).

Model 1: Unadjusted.

Model 2: Adjusted for demographic and clinical factors: age, sex, BMI, SBP, and UA.

Model 3: Adjusted for metabolic and familial factors: FBG, TG, TCHOL, and family history of hypertension.

Key findings: A consistent gradient of decreasing HRs was observed with lower eGFR quartiles (*P* < .0001 in all models). Compared to the highest eGFR quartile (Q1), the lowest quartile (Q4) showed the strongest association with the outcome (unadjusted HR = 0.09, 95% CI: 0.07–0.12; fully adjusted HR = 0.23, 95% CI: 0.17–0.31). The initially significant association in Q2 (unadjusted HR = 0.80, *P* = .0005) became nonsignificant after adjusting for confounders (model 3: HR = 0.99, *P* = .8753), indicating that demographic and metabolic factors substantially explained this association. These results confirmed the independent prognostic value of eGFR, with the strongest effects observed in the lowest eGFR quartile after a comprehensive adjustment for clinical confounders.

BMI = body mass index, CI = confidence interval, eGFR = estimated glomerular filtration rate, FBG = fasting blood glucose, HR = hazard ratio, SBP = systolic blood pressure, SD = standard deviation, TCHOL = total cholesterol, TG = triglycerides, UA = uric acid.

When eGFR was categorized into quartiles (Q1 as the reference), Q3 and Q4 were associated with markedly reduced hazards compared to Q1. In model 3, the HRs for Q2, Q3, and Q4 were 0.99 (95% CI: 0.87–1.13; *P* = .8753), 0.59 (95% CI: 0.49–0.70; *P* < .0001), and 0.23 (95% CI: 0.17–0.31; *P* < .0001), respectively, indicating a dose-dependent relationship in which higher eGFR was associated with a lower risk of the outcome. The *P* values for trend were < .0001 across all models, supporting a significant linear trend of decreasing hazard with increasing eGFR. These findings indicate that higher eGFR was independently associated with a lower risk of the outcome after adjustment for multiple cardiovascular and metabolic confounders.

### 3.3. Nonlinear eGFR-outcome relationships

RCS modeling was used to evaluate the association between eGFR and the primary outcome across the 3 sequentially adjusted Cox proportional hazards models (Fig. 2). All models demonstrated a significant overall association between eGFR and the primary outcome (all *P* < .001), firmly establishing eGFR as a robust predictor of outcome risk.

**Figure 2.**
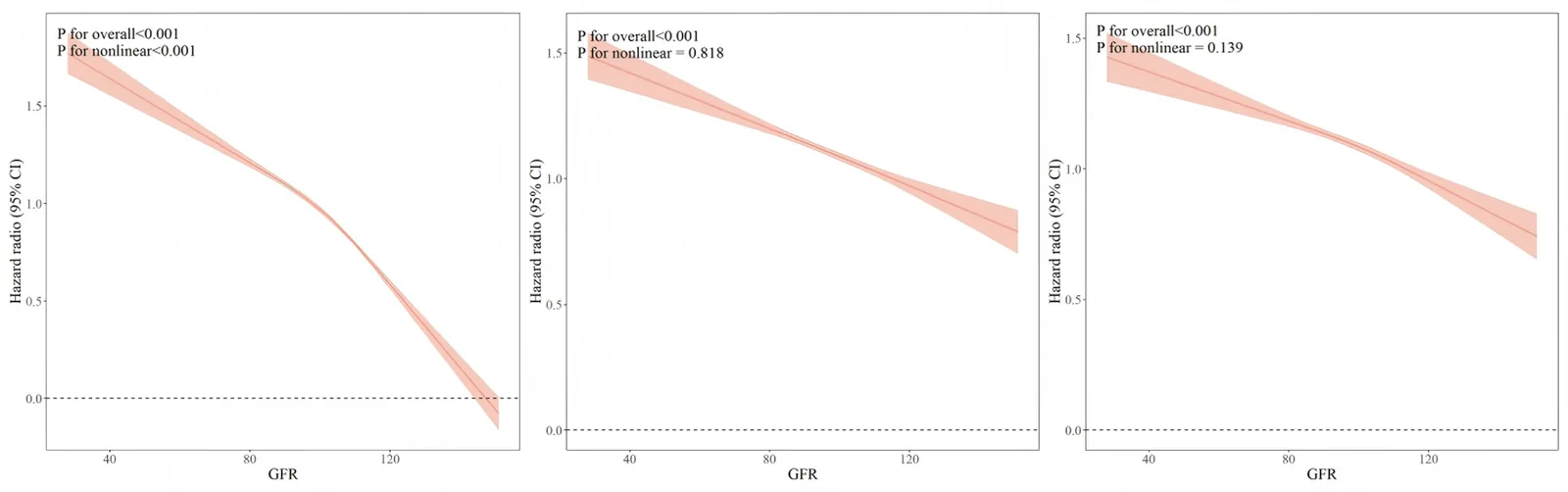
Restricted cubic spline plots depicting the association between eGFR and the HR for the primary outcome across 3 Cox proportional hazards models. The 3 panels (left to right) correspond to sequentially adjusted models. Model 1: unadjusted (no covariate adjustment). Model 2: Adjusted for demographic and clinical factors: age, sex, BMI, SBP, and UA. Model 3: Adjusted for metabolic and familial factors: FBG, TG, TCHOL, and family history of hypertension. Overall association: All models demonstrated a highly significant association between eGFR and primary outcome (*P* < .001), indicating that eGFR is a robust predictor of outcome risk. Nonlinearity testing: Model 1 showed a strong nonlinear trend (*P* < .001), with HR declining sharply as eGFR increased (consistent with the protective effect of higher renal function). In models 2 (*P* = .818) and 3 (*P* = .139), the nonlinear trend was substantially attenuated after adjusting for confounders, suggesting that demographic/clinical factors (model 2) and metabolic/familial factors (model 3) partially explained the nonlinear relationship observed in the unadjusted analysis. BMI = body mass index, eGFR = estimated glomerular filtration rate, FBG = fasting blood glucose, HR = hazard ratio, SBP = systolic blood pressure, TCHOL = total cholesterol, TG = triglyceride, UA = uric acid.

Regarding nonlinearity, model 1 (unadjusted) demonstrated a significant nonlinear relationship (*P* < .001), with HRs decreasing sharply as eGFR increased. Model 2, adjusted for demographic (age and sex) and clinical variables (BMI, SBP, and UA), demonstrated attenuation of the nonlinear relationship (*P* = .818). Model 3, which was further adjusted for metabolic factors (FBG, TG, and TCHOL) and parental history of HT, demonstrated additional attenuation of nonlinearity (*P* = .139). This adjustment left only a weak residual nonlinear association, suggesting that metabolic and familial factors partially accounted for the observed nonlinearity.

### 3.4. Subgroup analyses

Table 3 presents the subgroup-specific associations between eGFR groups and the FIB-4 index using multivariate Cox models. Model 1 remained unadjusted; model 2 adjusted for age, sex, UA, and BMI; and model 3 further adjusted for parental HT, SBP, DBP, TG, TCHOL, and FBG. Significant associations remained after full adjustment in both males (model 3: HR = 1.16; 95% CI: 1.02–1.32; *P**** ***= .0229) and females (model 3: HR = 1.32; 95% CI: 1.04–1.68; *P *= .0208). Participants aged 40 to 59 years demonstrated significant associations across all models (model 3: HR = 1.34; 95% CI: 1.18–1.53; *P* < .0001). Participants without a parental history of HT also retained significant associations in model 3 (HR = 1.23; 95% CI: 1.05–1.44; *P* = .0119). Normometabolic subgroups, including those with FBG < 7 mmol/L, BMI < 25 kg/m^2^, SBP < 140 mm Hg, TCHOL < 5.18 mmol/L, TG < 1.7 mmol/L, and UA < 420 μmol/L, consistently demonstrated significant associations in model 3 (all *P* < .05). Overall, the association between eGFR and the FIB-4 index remained significant across several demographic and metabolic subgroups.

**Table 3:** Multivariate-adjusted HRs for the association between GFR group and FIB-4 index across subgroups.

GFR Group	Participants	Model 1		Model 2		Model 3	
	n (%)	(HR, 95% CI)	*P*-value	(HR, 95% CI)	*P*-value	(HR, 95% CI)	*P*-value
Sex							
Male	3807 (75.4)	2.14 (1.91, 2.40)	<.0001	1.17 (1.03, 1.33)	.0163	1.16 (1.02, 1.32)	.0229
Female	1244 (24.6)	2.27 (1.81, 2.84)	<.0001	1.32 (1.04, 1.67)	.0228	1.32 (1.04, 1.68)	.0208
Age							
<40	2212 (43.8)	1.24 (0.50, 3.09)	.6362	1.26 (0.50, 3.14)	.6243	1.22 (0.48, 3.05)	.6761
≥40, <60	2528 (50.0)	1.32 (1.16, 1.50)	<.0001	1.37 (1.20, 1.56)	<.0001	1.34 (1.18, 1.53)	<.0001
≥60	311 (6.2)	0.97 (0.79, 1.20)	.8046	1.00 (0.79, 1.26)	.9847	0.99 (0.78, 1.26)	.9525
HT in parents, n (%)							
Neither parent	2797 (55.4)	2.24 (1.95, 2.58)	<.0001	1.22 (1.04, 1.43)	.0126	1.23 (1.05, 1.44)	.0119
At least 1 parent	2254 (44.6)	2.01 (1.73, 2.33)	<.0001	1.17 (0.99, 1.39)	.0702	1.16 (0.98, 1.38)	.0862
FBG group							
<7 mmol/L	4594 (91.0)	2.17 (1.94, 2.43)	<.0001	1.18 (1.04, 1.35)	.0094	1.16 (1.02, 1.32)	.0230
≥7 mmol/L	457 (9.0)	1.64 (1.30, 2.06)	<.0001	1.24 (0.96, 1.61)	.1040	1.29 (0.97, 1.70)	.0789
BMI (kg/m^**2**^)							
<25	1861 (36.8)	2.35 (1.98, 2.79)	<.0001	1.28 (1.06, 1.55)	.0122	1.27 (1.05, 1.54)	.0141
≥25, <30	2663 (52.7)	1.95 (1.70, 2.25)	<.0001	1.12 (0.95, 1.31)	.1777	1.11 (0.95, 1.30)	.1970
≥30	527 (10.4)	2.42 (1.81, 3.24)	<.0001	1.28 (0.88, 1.85)	.1935	1.28 (0.88, 1.85)	.1975
SBP group							
<140 mm Hg	4104 (81.3)	2.12 (1.87, 2.41)	<.0001	1.25 (1.08, 1.43)	.0019	1.24 (1.04, 1.48)	.0149
≥140 mm Hg	947 (18.7)	1.83 (1.54, 2.18)	<.0001	1.15 (0.94, 1.42)	.1813	1.16 (0.99, 1.35)	.0593
TCHOL group							
<5.18 mmol/L	2551(50.5)	2.24 (1.96, 2.56)	<.0001	1.16 (1.00, 1.35)	.0466	1.18 (1.02, 1.38)	.0301
≥5.18 mmol/L	2500(49.5)	1.95 (1.67, 2.28)	<.0001	1.21 (1.02, 1.43)	.0254	1.21 (0.98, 1.52)	.0793
TG group							
<1.7 mmol/L	2353 (46.6)	2.24 (1.96, 2.56)	<.0001	1.22 (1.03, 1.45)	.0218	1.24 (1.04, 1.48)	.0149
≥1.7 mmol/L	2698 (53.4)	1.95 (1.67, 2.28)	<.0001	1.18 (1.01, 1.37)	.0343	1.16 (0.99, 1.35)	.0593
UA group							
<420 μmol/L	3368 (66.7)	2.00 (1.76, 2.27)	<.0001	1.17 (1.02, 1.34)	.0237	1.17 (1.02, 1.34)	.0250
≥420 μmol/L	1683 (33.3)	2.63 (2.21, 3.15)	<.0001	1.23 (0.98, 1.53)	.0699	1.22 (0.98, 1.52)	.0793

Data are presented as hazard ratios (HRs) with 95% confidence intervals (CIs) estimated using Cox proportional hazards models for the association between estimated glomerular filtration rate (eGFR) group and the Fibrosis-4 (FIB-4) index across prespecified subgroups.

Model 1: Unadjusted.

Model 2: Adjusted for demographic and clinical factors: age, sex, BMI, SBP, and uric acid (UA).

Model 3: Adjusted for metabolic and familial factors: FBG, TG, TCHOL, and family history of hypertension.

Demographic factors.

Male sex showed a persistent association with higher risk (model 3: HR = 1.16; 95% CI: 1.02–1.32, *P *= .0229), while female sex trended similarly (HR = 1.32; 95% CI: 1.04–1.68, *P *= .0208).

Age ≥ 40 to <60 years was independently associated with increased risk (model 3: HR = 1.34; 95% CI: 1.18–1.53, *P *< .0001), whereas age ≥60 years showed no significant association.

Metabolic and cardiovascular factors.

Parents with HT showed a borderline association in the unadjusted analysis (model 1: HR = 2.01, *P *< .0001), which became nonsignificant after full adjustment (model 3: HR = 1.16, *P *= .0862).

FBG ≥ 7 mmol/L tended toward a higher risk (model 3: HR = 1.29; 95% CI: 0.97–1.70, *P *= .0789), with stronger unadjusted effects (model 1: HR = 1.64; *P *< .0001).

BMI < 25 kg/m^2^ and ≥30 kg/m^2^ showed persistent associations (model 3: HR = 1.27; 95% CI: 1.05–1.54, *P *= .0141 and HR = 1.28; 95% CI: 0.88–1.85, *P *= .1975, respectively), while the 25 to 30 kg/m^2^ group lost significance after adjustment.

SBP ≥ 140 mm Hg showed a trend toward significance (model 3: HR = 1.16; 95% CI: 0.99–1.35, *P *= .0593), with attenuated effects after confounder adjustment.

Lipid and uric acid markers.

TCHOL ≥ 5.18 mmol/L trended toward significance in model 3 (HR = 1.21; 95% CI: 0.98–1.52, *P *= .0793), while TG ≥ 1.7 mmol/L showed a similar pattern (HR = 1.16; 95% CI: 0.99–1.35, *P *= .0593).

UA ≥ 420 μmol/L showed strong unadjusted associations (model 1: HR = 2.63, *P *< .0001), which attenuated to borderline significance after adjustment (model 3: HR = 1.22, *P *= .0793).

These results indicate that demographic (age and sex) and obesity-related factors (BMI) exhibit more robust independent associations with the outcome, whereas metabolic markers (glucose, lipids, and UA) and hypertension show partially confounded effects.

BMI = body mass index, CI = confidence interval, eGFR = estimated glomerular filtration rate, FBG = fasting blood glucose, HR = hazard ratio, HT = hypertension, SBP = systolic blood pressure, TCHOL = total cholesterol, TG = triglycerides, UA = uric acid.

### 3.5. Mediating effect analysis

Figure 3 presents the mediation analysis evaluating the mediating role of UA in the correlation between eGFR and FIB-4 index. UA partially mediated this association, accounting for approximately 6.88% of the total effect. In addition, eGFR exerted a significant direct effect on the FIB-4 index (direct effect coefficient = −0.0056; *P* < .001), indicating that although UA mediated part of the eGFR-FIB-4 relationship, eGFR maintained a significant direct effect on the FIB-4 index independent of UA.

**Figure 3.**
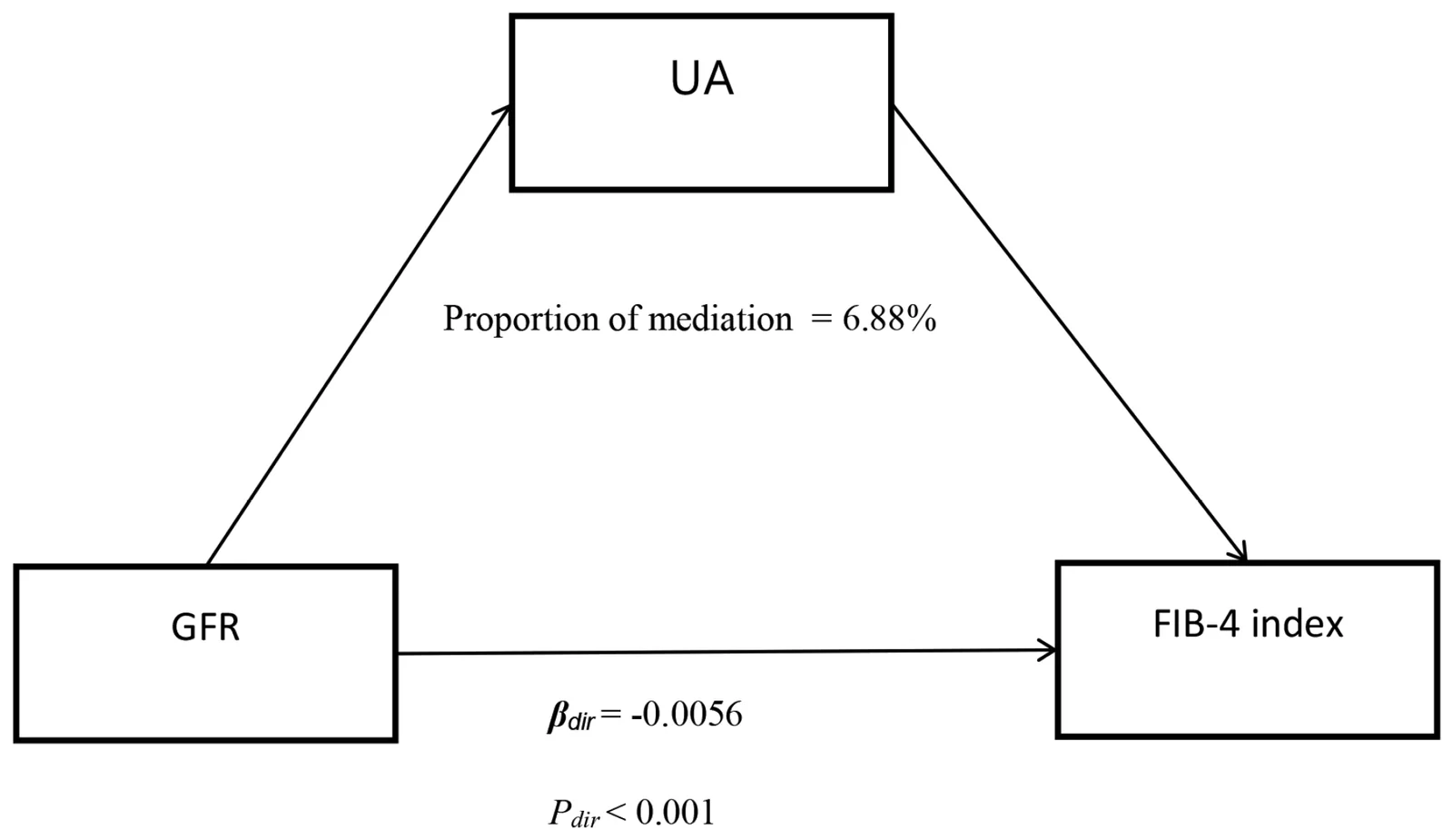
Mediation analysis of the effect of GFR on the FIB-4 index via UA. The results indicated that UA plays a mediating role in the relationship between GFR and the FIB-4 index, with a mediation proportion of 6.88%. The direct effect coefficient (β_dir) of GFR on the FIB-4 index was −0.0056, and the *P*-value for the direct effect (*P*_dir) was < .001, indicating a highly significant direct effect. FIB-4 index = Fibrosis 4 index, GFR = glomerular filtration rate, UA = uric acid.

## 4. Discussion

Our analysis of 5051 participants demonstrated a significant inverse relationship between eGFR and FIB-4 index. The HR for elevated FIB-4 levels declined with increasing eGFR, suggesting that better renal function is associated with a lower risk of liver fibrosis progression.

These findings are consistent with those of Kazuko Kotoku et al, who reported a progressive decline in eGFR accompanied by increasing FIB-4 indices across study groups, suggesting an association between declining kidney function and advancing liver fibrosis.^[26]^ Mechanistically, this relationship may be explained by shared pathological pathways, including chronic inflammation, oxidative stress, and UA-mediated crosstalk between the kidneys and liver.^[27,28]^ In the present study, UA partially mediated the association between eGFR and the FIB-4 index, accounting for 6.88% of the total effect. This finding suggests that hyperuricemia, which frequently accompanies reduced eGFR, may contribute to liver fibrosis.

The restricted cubic spline analysis further demonstrated a nonlinear relationship between eGFR and the FIB-4 index, indicating that the association between renal function and liver fibrosis may vary across different levels of renal function, particularly at lower eGFR values. This relationship may be influenced by chronic inflammation, systemic low-grade inflammation, and metabolic dysregulation.^[29-31]^ Subgroup analyses showed consistent inverse associations between eGFR and the FIB-4 index in males, females, middle-aged individuals, participants without a parental history of hypertension, and normometabolic subgroups, including those with normal fasting glucose, blood pressure, and lipid profiles. These findings suggest that the observed renal–hepatic association is consistent across multiple demographic and metabolic subgroups.^[32-34]^

Mediation analysis indicated that uric acid mediated 6.88% of the total effect, representing a statistically significant yet quantitatively modest indirect pathway. This finding points to a potential, though limited, involvement of uric acid metabolism in the renohepatic axis.^[35-38]^ However, the direct effect of eGFR on FIB-4 remained the dominant contributor to the overall association, underscoring its primary clinical relevance. These findings support an association between renal dysfunction and liver fibrosis and highlight the potential value of integrated renal and hepatic risk assessment. Given the high prevalence of CKD and nonalcoholic fatty liver disease, a better understanding of the mechanisms underlying this association may facilitate the development of integrated preventive and management strategies.^[39-41]^ The graded, dose-dependent association between eGFR quartiles and the risk of FIB-4 elevation observed in this study suggests the potential utility of a risk-stratified approach to liver fibrosis screening in clinical practice. Based on these findings, individuals with eGFR ≥ 90 mL/min/1.73 m^2^ may be considered at relatively low risk and may benefit from routine FIB-4 assessment during standard health examinations, particularly in the presence of conventional metabolic risk factors such as obesity, diabetes, or dyslipidemia. Individuals with eGFR of 60 to 89 mL/min/1.73 m^2^ may warrant closer monitoring of FIB-4 together with metabolic parameters, including UA, BMI, and FBG. This group, corresponding to KDIGO G2 stage, may also benefit from lifestyle interventions, such as dietary modification, increased physical activity, and weight management, to reduce metabolic risk and help preserve renal function. When hyperuricemia (UA ≥ 420 μmol/L) coexists with mildly reduced eGFR, further clinical evaluation may be warranted in accordance with current clinical guidelines. Individuals with eGFR < 60 mL/min/1.73 m^2^, corresponding to KDIGO G3a–G5 stages, demonstrated the greatest risk of FIB-4 elevation and may benefit from closer surveillance and comprehensive assessment of metabolic risk factors, including UA, BMI, FBG, lipid profile, and blood pressure. In such individuals, multidisciplinary management involving nephrology and hepatology services may facilitate comprehensive evaluation and coordinated care. Overall, these findings support the potential value of integrating renal function and metabolic assessment into liver fibrosis risk stratification. However, prospective studies are needed to validate these observations and determine the optimal screening strategies and management approaches for individuals at increased risk of liver fibrosis.

This study has several limitations. First, its observational design inherently precludes strong causal inferences. Second, despite our subgroup analyses, other potential confounding factors may have been overlooked. For example, medication use (e.g., urate-lowering agents and antihypertensive drugs), dietary patterns, alcohol consumption history, confirmed type 2 diabetes, the severity of hepatic steatosis, and physical activity levels were not included in the analysis, which may have affected the precision of the observed associations. Third, the sample size in key subgroups was limited. In this study, the eGFR < 60 mL/min/1.73 m^2^ group comprised only 30 participants (0.6%). Although this low proportion is consistent with the true prevalence of moderate-to-severe renal dysfunction in the general health checkup population, the small sample size inevitably led to wide confidence intervals around the point estimates, limiting the precision and reliability of the observed effect sizes in this subgroup. Therefore, the conclusions regarding the relationship between moderate-to-severe renal dysfunction and liver fibrosis warrant further validation in larger, independent cohorts enriched with patients with chronic kidney disease. Fourth, the development and progression of liver fibrosis typically require several years or even decades, and the current observation period of 12.6 months may have been insufficient to capture the delayed and cumulative effects of declining eGFR on liver fibrosis progression. Longer follow-up will facilitate a more accurate assessment of the long-term impact of sustained eGFR reduction on hepatic structural and functional changes.

Overall, this study demonstrated that a lower eGFR is independently associated with an increased FIB-4 index, reflecting a nonlinear relationship whereby renal dysfunction is associated with more severe liver fibrosis. This association remained robust across diverse demographic and metabolic subgroups, with UA partially mediating the effect of eGFR on the FIB-4 index. These findings underscore the vital interconnection between renal and hepatic health and suggest that eGFR may serve as a useful marker for liver fibrosis assessment in clinical practice. Given the substantial global burden of CKD and liver fibrosis, these findings support the need for integrated renal–hepatic screening strategies, particularly in individuals with metabolic comorbidities. Future studies should further investigate the underlying causal mechanisms and long-term outcomes to inform targeted interventions.

## Acknowledgments

The authors thank all participants and staff involved in this study for their invaluable contributions.

## Author contributions

**Conceptualization:** Weina Xu.

**Data curation:** Danxiang Chen, Saiyun Lou, Jiannan Wu, Ruiqi Liu, Hanjie Chen.

**Formal analysis:** Weina Xu, Danxiang Chen, Ruiqi Liu.

**Funding acquisition:** Weina Xu.

**Methodology:** Danxiang Chen.

**Project administration:** Weina Xu.

**Resources:** Jiannan Wu, Yinshan Liu

**Software:** Yinshan Liu, Hanjie Chen

**Validation:** Saiyun Lou, Yinshan Liu

**Visualization:** Yinshan Liu

**Writing – original draft:** Danxiang Chen.

**Writing – review & editing:** Weina Xu, Saiyun Lou.
